# Vascular Comorbidities and an Increased Comorbidity Score Are Associated With Disability and Disability Progression in Secondary Progressive Multiple Sclerosis

**DOI:** 10.1111/ene.70517

**Published:** 2026-02-09

**Authors:** Nevin A. John, Yingtong Li, Floriana De Angelis, Ferran Prados Carrasco, Jon Stutters, Anisha Doshi, Alberto Calvi, Domenico Plantone, Thomas Williams, Thanh Phan, Jeremy Chataway, for the MS‐SMART Investigators, Sebastien Ourselin, Marie Braisher, Tiggy Beyene, Vanessa Bassan, Alvin Zapata, Siddharthan Chandran, Peter Connick, Dawn Lyle, James Cameron, Daisy Mollison, Shuna Colville, Baljean Dhillon, Moira Ross, Gina Cranswick, Allan Walker, Lorraine Smith, Gavin Giovannoni, Sharmilee Gnanapavan, Richard Nicholas, Waqar Rashid, Julia Aram, Helen Ford, Sue H. Pavitt, James Overell, Carolyn Young, Heinke Arndt, Martin Duddy, Joe Guadagno, Nikolaos Evangelou, Matthew Craner, Jacqueline Palace, Jeremy Hobart, Basil Sharrack, David Paling, Clive Hawkins, Seema Kalra, Brendan McLean, Nigel Stallard, Roger Bastow

**Affiliations:** ^1^ Department of Medicine School of Clinical Sciences, Monash University Clayton Victoria Australia; ^2^ Department of Neurology Monash Health Melbourne Australia; ^3^ Department of Neuroinflammation, Queen Square Multiple Sclerosis Centre UCL Institute of Neurology, Faculty of Brain Sciences, University College London London UK; ^4^ Centre for Medical Image Computing (CMIC), University College London London UK; ^5^ e‐Health Center, Universitat Oberta de Catalunya Barcelona Spain; ^6^ Laboratory of Advanced Imaging in Neuroimmunological Diseases (imaginEM), Fundació de Recerca Clínic Barcelona – Institut d'Investigacions Biomèdiques August Pi I Sunyer (FRCB‐IDIBAPS) Barcelona Spain; ^7^ Department of Medicine, Surgery & Neuroscience University of Siena Siena Italy; ^8^ National Institute for Health Research (NIHR), University College London Hospitals (UCLH) Biomedical Research Centre (BRC) London UK

**Keywords:** comorbidities, multiple sclerosis, secondary progressive

## Abstract

**Background:**

Vascular risk factors are associated with increased disease activity and disability progression in multiple sclerosis (MS). This has been studied mainly in cohorts with relapsing–remitting MS. However, the association between vascular comorbidities (VCM) and clinical disability in secondary progressive MS (SPMS) is less well studied. Our aim was to investigate the association between VCM, non‐VCM, comorbidity burden and both physical and cognitive performance in SPMS.

**Methods:**

Longitudinal analysis of 445 patients from the MS secondary progressive multi‐arm trial (MS‐SMART)–a multi‐arm multicentre phase‐2b randomised placebo‐controlled trial of three agents in SPMS (NCT01910259). VCM (hypertension and hyperlipidaemia) and non‐VCM (asthma, hypothyroidism and osteoporosis) were recorded. A comorbidity score was also determined (0, 1, ≥ 2). Physical disability and processing speed were assessed at baseline, 48‐ and 96 weeks. Multiple linear regression and mixed models were used to investigate the cross‐sectional and longitudinal relationships between baseline VCM, non‐VCM, comorbidity score and clinical outcome measures.

**Results:**

The cohort was predominantly female (67%), median Expanded Disability Status Scale (EDSS) 6.0. 13% and 9% had hypertension and hyperlipidaemia (VCM), respectively. 7%, 9% and 5% had asthma, hypothyroidism and osteoporosis (non‐VCM), respectively. Co‐morbidity counts were 0,63%; 1, 23% and with > = 2, 11%. In cross‐sectional models, both hypertension (*β* = 0.36, 95% CI 0.18–0.54) and an increased comorbidity count (*β* = 0.47, 95% CI 0.28–0.67) were associated with higher EDSS scores. In longitudinal models, hyperlipidaemia (*β* = 0.22, 95% CI 0.02–0.42) and increased comorbidity count (*β* = 0.21, 95% CI 0.01–0.41) were associated with increased EDSS scores over 48/96 weeks. No associations were seen with the non‐VCM.

**Conclusion:**

VCM and also increased comorbidity burden per se are associated with increased disability. Disability worsening over 96 weeks was most evident in those with hyperlipidaemia and increased comorbidity burden.

## 1 Introduction

The importance of comorbidities in MS is increasingly recognised and may present an adjunctive therapeutic strategy to decrease disability progression. With the increasing age of those with secondary progressive MS (SPMS) compared to earlier, more relapsing forms, comorbidities, and in particular overall cardiovascular risk, are likely to be greater in those with SPMS [1, 2]. Comorbidities in MS are associated with physical and cognitive disability, increased risk of disease worsening, decreased quality of life and increased utilisation of health resources [2, 3]. Thus far, studies have been mostly undertaken in large registries or used hospital admission statistics, often using self‐reported or patient reported measures in cohorts containing predominantly earlier relapsing forms of MS [4, 5, 6, 7, 8, 9, 10, 11, 12]. The impact of comorbidities on disability in progressive forms of MS has been less well studied.

The Multiple Sclerosis—Secondary Progressive multi‐arm trial (MS‐SMART) is a phase 2b multi‐arm multicentre, randomised placebo‐controlled trial of the neuroprotective potential of amiloride, fluoxetine and riluzole in SPMS (NCT01910259) [13]. The trial did not demonstrate any effect of the treatment agents on decreasing whole brain atrophy over 96 weeks in SPMS [14]. A recent analysis of this SPMS cohort (*n* = 445) published in this journal demonstrated that vascular comorbidities (VCM) were associated with grey matter atrophy [15].

We now examine the effect of (i) individual vascular comorbidities (VCM) (ii) non‐vascular comorbidities (non‐VCM) and (iii) increasing comorbidity burden on measures of ambulation, upper limb function and cognitive performance, both at baseline and longitudinally for the duration of the study.

## 2 Methods

### 2.1 Study Participants

In brief, participants were eligible for this trial if aged 25–65 with an Expanded Disability Status Scale (EDSS) score of 4.0–6.5 and demonstrated evidence of progression of SPMS over the last 2 years. Further details of the study protocol including complete eligibility criteria, primary and secondary outcome measures have been described previously [13, 15].

Consent was obtained for all participants according to the Declaration of Helsinki and ethical approval for the study was provided by the Scotland A Research Ethics Committee [13/SS/0007].

### 2.2 Comorbidity Status

The presence of VCM (hypertension, hyperlipidaemia) and non‐VCM (asthma, hypothyroidism, osteoporosis) was recorded at baseline. Comorbidity count was also determined (0, 1, 2 or more). These were recorded from participant histories and corroborated with GP and hospital records where possible. Where any of the comorbidities were recorded at screening, it was assumed to be present for the entire trial duration.

### 2.3 Clinical Measures of Disability

Participants underwent a series of clinical assessments at baseline, 48 and 96 weeks (end of study). The Expanded Disability Status Scale (EDSS) and timed 25‐ft walk (T25FW) were used to measure disability and walking speed, respectively [16, 17]. Upper limb function was measured using the 9‐Hole peg test (9HPT), and processing speed performance was assessed using the symbol digit modalities test (SDMT) [18, 19].

### 2.4 MRI Analysis

These have been described in detail previously but in brief, standardised MRI acquisitions were taken at 3 Tesla [13, 15]. Acquisition parameters used in the study are shown in Table S1. T2 lesion volume was calculated using a semi‐automated method with Jim7 (Xinapse, UK); SIENAX was used to measure normalised brain volume [15, 20].

### 2.5 Statistical Analysis

Participant demographics, VCM, non‐VCM frequency and clinical outcome measures were summarised using descriptive statistics. Missing data are reported but not imputed. Baseline comorbidities were analysed as (i) individual components recorded as binary (present/absent) variables; and (ii) comorbidity count (0, 1, 2 or more) to enable comparison with previous studies (categorical variable with no comorbidities as reference) [7]. The comorbidity count was truncated at two or more due to the low number of participants with 3 or more total comorbidities.

In the baseline cross‐sectional analysis, the dependent variables were EDSS, T25FW, 9HPT, SDMT (all continuous).

In stage 1 analyses, we used multiple linear regression models to explore the relationship VCM/non‐VCM, comorbidity count and baseline disability outcome measures. The dependent variable was the baseline clinical measure, and the independent variable of interest was the presence/absence of the individual VCM/non‐VCM or comorbidity count. 95% confidence intervals for regression coefficients were computed using percentile bootstrap confidence intervals based on 10,000 bootstrap samples.

In the second stage analysis, linear mixed models with robust standard errors were used to explore the relationship between the respective baseline VCM or comorbidity count and the change in disability over 48 and 96 weeks measured using the change in the clinical outcome measure from baseline to 48 and 96 weeks. Models were adjusted for age (continuous), sex (female as reference), ethnicity (white Caucasian as reference), disease duration (continuous), treatment allocation (for models examining change over 48/96 weeks only), BMI (categorised as follows: underweight < 18.5 kg/m^2^, normal [reference] 18.5–24.9 kg/m^2^, overweight 25.0–29.9 kg/m^2^ and obese ≥ 30 kg/m^2^), baseline normalised brain volume (continuous) and T2LV (continuous). Sensitivity analyses were performed to investigate the effect of an interaction with time, exclusion of T2LV and inclusion of study site as covariates. At the reviewers' request, further sensitivity analyses were also performed excluding the amiloride treatment group, due to potentially confounding effects of amiloride on hypertension.

We also examined the relative contribution of the relevant VCM/non‐VCM (or comorbidity count) and each covariate in the regression models to clinical disability or disability progression by calculating Shapley Additive exPlanations (SHAP) values. Analysis using SHAP values is based on cooperative game theory [21]. The Shapley value is measured as the average of all permutations of the coalition of the covariates containing the covariate of interest minus the coalition without the covariate of interest [10]. In the context of regression, it assigns an importance value to each regressor, which represents the additive marginal effect on the model prediction of including that regressor.

Results from the regression models are reported as standardised coefficients (*β*) with 95% confidence intervals. Distributional assumptions in the regression analyses were assessed by visual inspection of residual plots, normality examined by normal probability plots and highly leveraged data observations identified using Cook's distance. Multi‐collinearity was examined using variable inflation factors.

Statistical analysis was completed using R statistical software version 4.0.3 (https://www.r‐project.org) and Stata version 18 (https://www.stata.com) [22]. All statistical tests and confidence intervals were two‐sided. 95% confidence intervals were calculated with the significance of raw *p*‐values assessed based on a 5% significance level. No adjustment for multiplicity was made as comparisons were based on specific a priori hypotheses.

## 3 Results

### 3.1 Participants

445 participants were randomised across 13 centres. Cohort demographics were compatible with a typical population of SPMS, with a mean age 54.6 (SD 7.0), 67% female and a median EDSS of 6.0 (range 4.0–6.5) (Table 1). 62/445 (14%) did not undergo clinical assessment with EDSS at 96 weeks. This group had similar characteristics to the analysed cohort. The percentage loss to follow‐up was 5%–9% across all study groups.

**TABLE 1:** Baseline demographics and characteristics of the MS‐SMART cohort.

Characteristic	*N* = 445
Age (years), mean (SD)	54.5 (7.0)
Females, *n* (%)	298 (67%)
Ethnicity, *n* (%)	
White	427 (96%)
Black	5 (1%)
Asian	10 (2%)
Other	3 (0.7%)
Disease duration in years, mean (SD)	21.6 (9.7)
Any previous use of disease modifying therapy	160 (36%)
Expanded Disability Status Scale, median (range)	6.0 (4.0–6.5)
Timed 25Ft walk (sec), median (IQR)	11.3 (10.2)
9‐hole peg test (sec‐1), mean (SD)	0.03 (0.01)
Symbol digit modalities test, median (IQR)	46 (17)
Normalised whole brain volume (mL), mean (SD)	1422.6 (83.6)
T2 lesion volume (mL), median (IQR)	10.3 (14.5)
Individual comorbidities, *n* (%)	
Asthma	31 (7%)
Hypertension	60 (13%)
Hyperlipidaemia	41 (9%)
Osteoporosis	22 (5.0%)
Hypothyroidism	41 (9%)
Body mass index category, *n* (%)	
Underweight	22 (5%)
Healthy	204 (46%)
Overweight	136 (31%)
Obese	81 (18%)
Comorbidity count, *n* (%)	
0 comorbidities	279 (63%)
1 comorbidities	115 (26%)
≥ 2 comorbidities	51 (11%)

166/445 (37%) had at least one VCM with 13% having hypertension and 9% with hyperlipidaemia. For non‐VCM, 5%, 7% and 9% had osteoporosis, asthma and hypothyroidism, respectively. When examining characteristics by cumulative number of comorbidities, 63% had none, 26% had one and 11% had two or more (Table 1).

### 3.2 VCM Analyses

**FIGURE 1:**
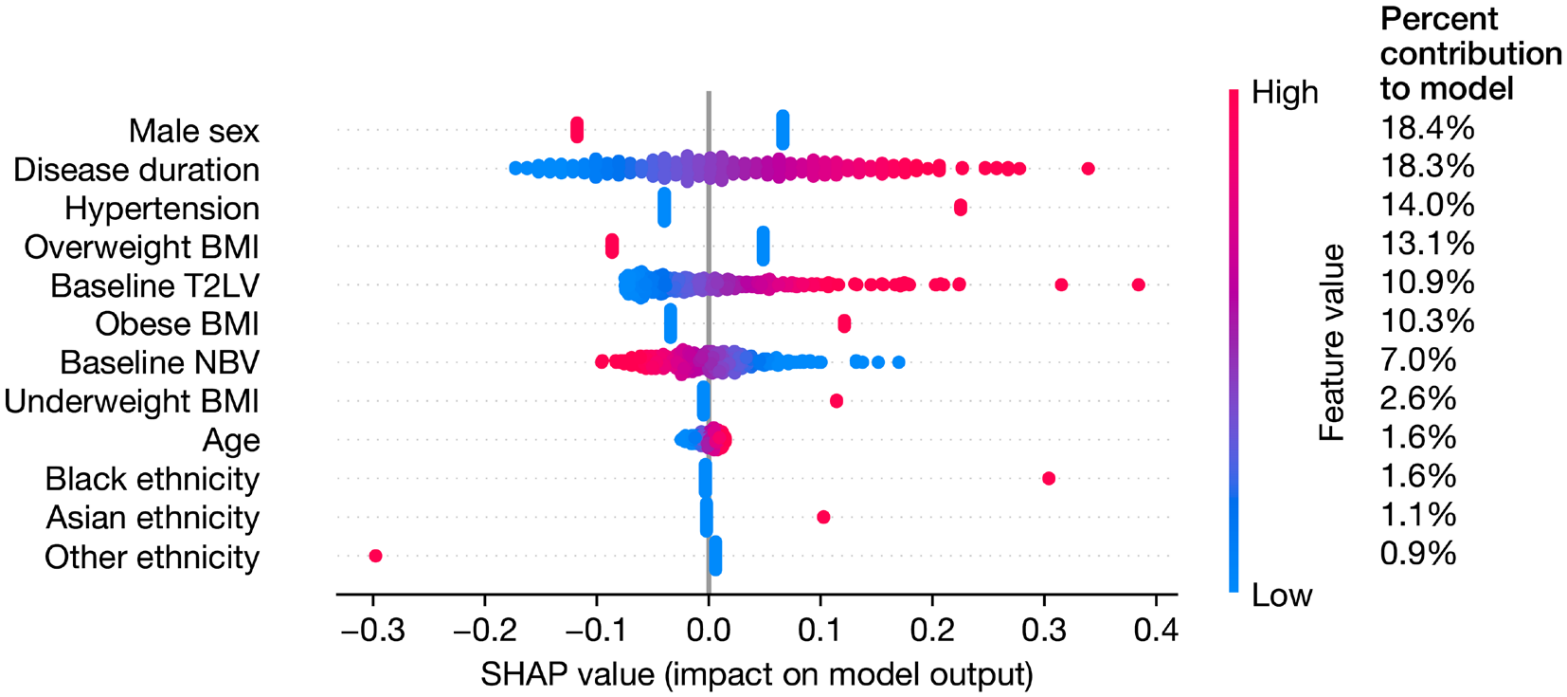
SHAP analysis of association between hypertension and EDSS scores at baseline. SHAP values for parameters in the multiple linear regression evaluating the association between hypertension and baseline EDSS. The column on the right shows the mean absolute SHAP value for each parameter, as a percentage of the total, representing the percent contribution of each parameter to the model output. The *x*‐axis shows the SHAP values, with each individual dot signifying the SHAP value of a particular feature for a given data point. BMI, body mass index; EDSS, Expanded Disability Status Scale; SHAP, SHapley Additive exPlanations; T2LV, T2 lesion volume.

In the step 1 regression analysis, the presence of hypertension at baseline (*β* = 0.36, bootstrapped 95% CI 0.18–0.54, *p* < 0.001) was associated with increased EDSS scores at baseline. In this model, being male was also associated with an EDSS score that was 0.25 standard deviations lower than females. There was no association with age (Table 2). From the SHAP analysis, the model contribution of hypertension was ~14% behind only disease duration and being male in importance (Figure 1). There was no association between the presence of hypertension and change in EDSS scores over 48 and 96 weeks.

**TABLE 2:** Association between hypertension and EDSS at baseline.

Predictors	Beta	Bootstrapped 95% CI	*p*
Hypertension	0.36	0.18 to 0.54	**< 0.001**
Male sex	−0.25	−0.46 to −0.04	**0.02**
Disease duration	0.13	0.05 to 0.22	**0.001**

*Note:* *R* ^2^ = 0.10. Other non‐significant model covariates—age, ethnicity, normalised brain volume, T2 lesion volume and body mass index are not shown. Bold values: Hypertension, disease duration were associated with increased EDSS scores. Male sex was associated with decreased EDSS scores.

**FIGURE 2:**
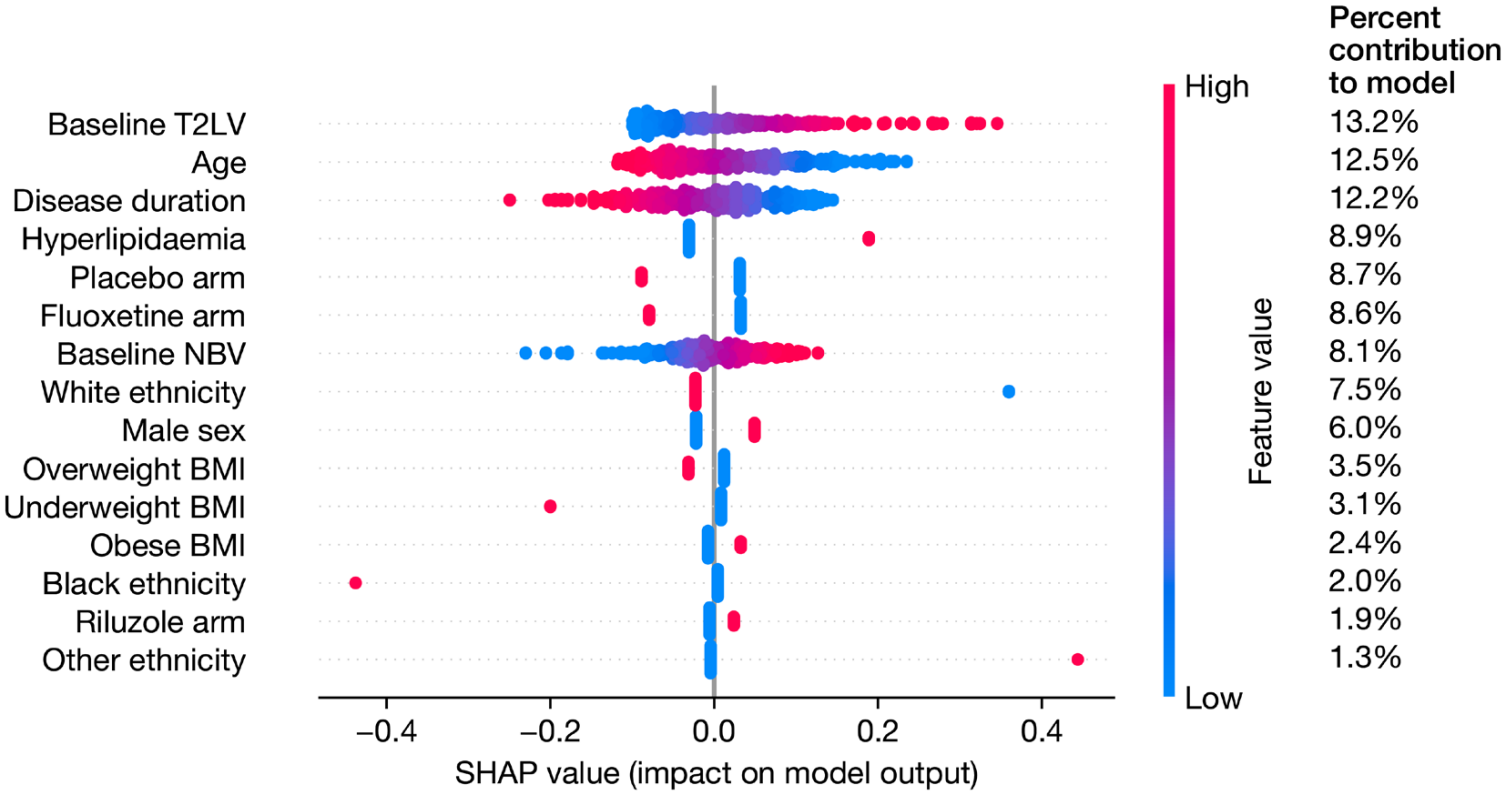
SHAP analysis of association between hyperlipidaemia and EDSS change over 48/96 weeks. SHAP values for parameters in the linear mixed model evaluating the association between hyperlipidaemia and change in EDSS over 48/96 weeks. The column on the right shows the mean absolute SHAP value for each parameter, as a percentage of the total, representing the percent contribution of each parameter to the model output. The *x*‐axis shows the SHAP values with each individual dot signifying the SHAP value of a particular feature for a given data point. BMI, body mass index; EDSS, Expanded Disability Status Scale; NBV, normalised brain volume; SHAP, SHapley Additive exPlanations; T2LV, T2 lesion volume.

The presence of hyperlipidaemia at baseline (*β* = 0.22, 95% CI 0.02–0.42, *p* = 0.04) was associated with a greater increase in EDSS scores over 48 and 96 weeks (Table 3). Age and sex were not associated with a change in EDSS scores over 96 weeks. In the SHAP analysis, this contributed 8.9% to the model and was third in importance to T2 lesion volume, age and disease duration (Figure 2). There was no interaction effect with time (Table S1). The association remained if the amiloride group were excluded (Table S2). The effect size and confidence intervals were very similar in the sensitivity analyses excluding T2LV or including study site (Tables S3 and S4), though fell marginally outside the significance threshold.

**TABLE 3:** Association between hyperlipidaemia and EDSS change over 48/96 weeks.

Predictors	Beta	Standardised 95% CI	*p*
Hyperlipidaemia	0.22	0.02 to 0.42	**0.035**
Other ethnicity	0.45	0.02 to 0.87	**0.039**
T2 lesion volume	0.10	0.02 to 0.18	**0.011**
Disease duration	−0.08	−0.15 to −0.01	**0.041**

*Note:* *n* = 362. Other non‐significant model covariates—age, sex, normalised brain volume, body mass index and treatment allocation are not shown.

Bold values: Hypertension, disease duration were associated with increased EDSS scores. Male sex was associated with decreased EDSS scores.

No other associations were seen between the individual VCM (hypertension, hyperlipidaemia), ambulation (T25FW), upper limb function (9HPT) or cognition (SDMT).

### 3.3 Non‐VCM Analyses

No associations were seen between the non‐VCM and EDSS, T25FW, 9HPT and SDMT.

### 3.4 Comorbidity Count

Having two or more comorbidities was associated with increased EDSS scores (*β* = 0.47, bootstrapped 95% CI 0.28–0.67, *p* < 0.001) and T25FW values (*β* = −0.34, 95% CI [−0.69 to 0.00], *p* = 0.049) at baseline (Table 4, Table S5). Being male was also associated with an EDSS that was 0.24 standard deviations lower than females; however, age did not show a significant association (Table 4). The SHAP analysis showed that having two or more comorbidities contributed 15% to the model, around the same as disease duration (17%) and male sex (17%) (Figure S1).

**TABLE 4:** Association between comorbidity count and EDSS at baseline.

Predictors	Beta	Bootstrapped 95% CI	*p*
One comorbidity	0.06	−0.15 to 0.27	0.58
≥ 2 or more comorbidities	0.47	0.28 to 0.67	**< 0.001**
Male sex	−0.24	−0.44 to −0.03	**0.03**
Disease duration	0.13	0.04 to 0.21	**0.005**

*Note:* *n* = 433, *R* ^2^ = 0.10. Other non‐significant model covariates—age, ethnicity, normalised whole brain volume, T2 lesion volume and body mass index are not shown.

Bold values: Hypertension, disease duration were associated with increased EDSS scores. Male sex was associated with decreased EDSS scores.

**FIGURE 3:**
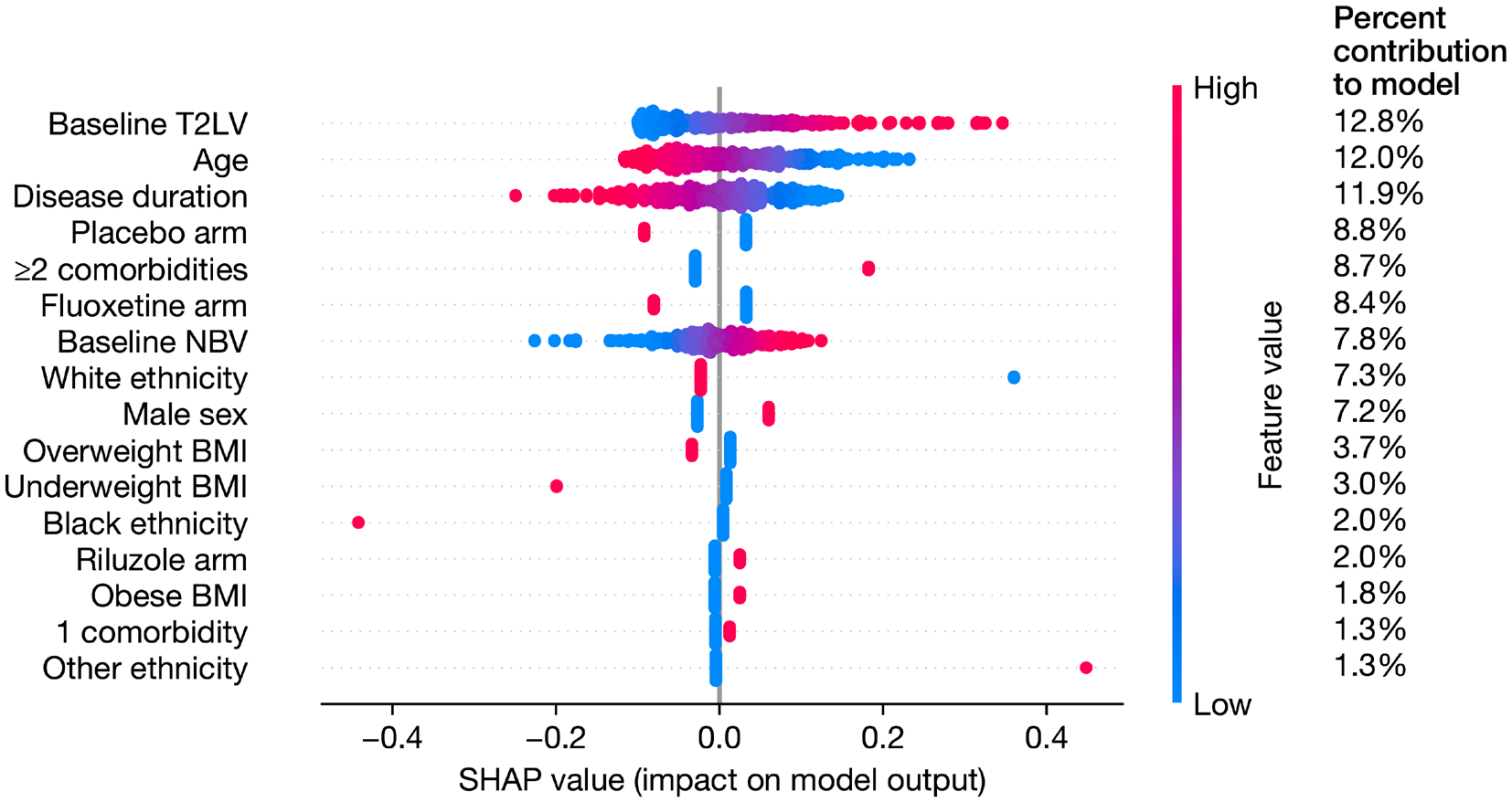
SHAP analysis of association between comorbidity count and EDSS change over 48/96 weeks. SHAP values for parameters in the multiple linear regression evaluating the association between comorbidity count and change in EDSS over 48/96 weeks. The column on the right shows the mean absolute SHAP value for each parameter, as a percentage of the total, representing the percent contribution of each parameter to the model output. The *x*‐axis shows the SHAP values with each individual dot signifying the SHAP value of a particular feature for a given data point. BMI, body mass index; EDSS, Expanded Disability Status Scale; NBV, normalised brain volume; SHAP, SHapley Additive exPlanations; T2LV, T2 lesion volume.

Having 2 or more comorbidities was also associated with a change in EDSS from baseline over 48 and 96 weeks (*β* = 0.21, 95% CI 0.01–0.41, *p* = 0.04) (Table 5). Age and sex were not associated with a change in EDSS scores over 96 weeks in this model. In the SHAP analysis, having 2 or more comorbidities contributed 8.7% to the model, and in terms of feature importance, was lower than T2 lesion volume, age, disease duration and treatment arm (Figure 3). There was no interaction effect with time (Table S6). The association remained if the amiloride group were excluded (Table S7). The effect size and confidence intervals were robust in the sensitivity analyses excluding T2LV or including study site (Tables S8 and S9); the association remained significant when adjusted for study site (Table S9).

**TABLE 5:** Association between comorbidity count and EDSS change over 48/96 weeks.

Predictors	Beta	Standardised 95% CI	*p*
One comorbidity	0.02	−0.15 to 0.18	0.835
Two or more comorbidities	0.21	0.01 to 0.41	**0.037**
Other ethnicity	0.45	0.03 to 0.87	**0.036**
T2 lesion volume	0.10	0.02 to 0.18	**0.011**
Disease duration	−0.08	−0.15 to −0.01	**0.044**

*Note:* *n* = 362. Non‐significant model covariates—age, sex, treatment allocation, normalised whole brain volume and body mass index are not shown.

Bold values: Hypertension, disease duration were associated with increased EDSS scores. Male sex was associated with decreased EDSS scores.

## 4 Discussion

In our analysis of a large SPMS randomised control trial cohort followed up for 2 years, we demonstrate that both hypertension, hyperlipidaemia and an increasing absolute comorbidity burden are associated with increased physical disability in SPMS.

### 4.1 Frequency of Comorbidities in SPMS

Hypertension was the most common VCM, followed by hyperlipidaemia. Making comparisons with previous prevalence studies is challenging due to heterogeneous MS cohorts, variable methods when estimating prevalence and a relative paucity of population‐based studies in cohorts with SPMS. However, our findings are generally in keeping with estimates that have been reported in the literature for hypertension (10%–19%), hyperlipidaemia (11%), asthma (7%) and thyroid disease (6%) including those from a recent meta‐analysis of pooled clinical trial data [2, 3, 23]. Given the older age of the cohort, we would expect there to be higher percentage with comorbidities reflected by the fact that 37% of participants had at least 1 or more comorbidity.

### 4.2 Vascular Comorbidities and Physical Disability

Our findings suggest that the presence of hypertension is associated with increased physical disability as reflected by EDSS in SPMS. After adjusting for covariates, those with hypertension have an EDSS that is 0.36 standard deviations greater than those without hypertension. The model explained 10% of the variation in EDSS suggesting that there are unexplained factors not captured in the model. When exploring the importance or contribution of each variable in the model using SHAP analysis, hypertension showed a greater contribution than T2LV, but disease duration and being male had a greater contribution to increased EDSS scores at baseline. This may reflect previous studies that male sex is associated with an increased risk of progression, and limited associations between lesion load and disability progression once in the secondary progressive phase of MS [24]. Previous studies have examined mixed cohorts containing predominantly relapsing MS in prospective and retrospective patient registries. Using the patient reported NARCOMS registry, Marrie et al. demonstrated that those with hypertension were more likely to develop ambulatory disability (EDSS 4, 6 or 6.5) [11]. However, the disease course was not specified which limits the direct generalisability to SPMS. Conway et al. showed that hypertension was associated with slower walking speed (T25FW), but this was in a study of 2083 people with RRMS [8]. In a 20 year retrospective analysis of 2396 patients, of which 21.1% had PPMS or SPMS, Dagan et al. showed that those with a history of hypertension were more likely to reach EDSS 4, 6 or 8 compared to those without [25]. However, a Canadian retrospective analysis of 3166 (8.6% PPMS) did not find an association with EDSS progression in adjusted models [26]. In prospective studies, Kowalec et al. showed hypertension was not associated with increased relapse risk over 2 years, but this cohort (*n* = 885) contained only 22% with SPMS with a baseline median EDSS of 2.5 [7].

We also identified an association between hyperlipidaemia on physical disability progression over 96 weeks in SPMS. Previous studies that included progressive MS (PMS) have shown mixed results. Fitzgerald et al. in a cross‐sectional study showed dyslipidaemia was associated with worse upper limb function and processing speed (*n* = 11,507, PMS 29%; EDSS not studied) [27]. Zhang et al. in a retrospective study showed no association between hyperlipidaemia and EDSS (*n* = 3166, PMS 9%) [26]; whilst a prospective study by Kowelec et al. (see above) showed hyperlipidaemia to be associated with increased relapse risk but disease progression was not investigated [7]. However, these cohorts contained predominantly relapsing remitting forms, were either cross‐sectional or did not include follow‐up data on disease progression thereby limiting comparison with our study that contained progressive inactive SPMS participants followed up within an RCT over 96 weeks.

Building upon this previous work but in a large cohort with secondary progressive disease course, our study demonstrates the association between hypertension, hyperlipidaemia and increased disability (as reflected by EDSS) in an SPMS cohort showing evidence of disease progression before and during the trial.

### 4.3 Vascular Comorbidities and Cognitive Performance

VCM were not associated with decreased performance on tests of cognitive processing speed in this study. Previous studies have shown mixed results—a propensity weighted retrospective study of the MS‐PATHS registry (*n* = 11,507, 26% progressive disease course) demonstrated those with 2 or more comorbidities had decreased processing speed performance assessed using MS performance test (MSPT) [27]. Abbatemarco et al. studied 4344 patients (19.7% SPMS) showing that VCM burden was associated with decreased performance on processing speed (assessed using MSPT); whilst Marrie et al. showed that diabetes mellitus was associated with decreased performance on BVMT‐R results in a cohort of 111 (12% SPMS) [4, 28]. However, these studies were undertaken in cohorts with predominantly relapsing remitting MS using slightly different methods to measure processing speed. Recently published cross‐sectional data from a cohort of SPMS (*n* = 218) recruited for the MS‐STAT2 trial (NCT03387670) showed that prematurely achieved vascular risk measured using QRISK3 was associated with decreased performance on verbal memory (CVLT‐II) [29]. Further analysis using additional cognitive domains in SPMS is needed and is being conducted in current clinical trials such as MS‐STAT2 (NCT03387670).

### 4.4 Non‐Vascular Comorbidities and Disability Measures

Our findings are generally in keeping with previous studies which did not show any association between asthma and time to EDSS or patient reported outcome measures [30]. A recent meta‐analysis examining comorbidities reported in MS clinical trials found mixed findings with ‘lung conditions’ and ‘autoimmune thyroid disease’ associated with disability worsening but not disease activity. However, again the majority were RRMS studies.

### 4.5 Comorbidity Score and Disability

Having two or more comorbidities at baseline was associated with increased clinical disability and decreased ambulatory function as reflected by EDSS and T25FW, respectively. In terms of model importance, only being male (mean absolute SHAP value 17%) and longer disease duration (17%) had more contribution to increased physical disability at baseline. The relationship between increasing comorbidity burden and ambulatory function has been previously demonstrated where Marrie et al. showed that the addition of a comorbidity was associated with 13%–18% increased odds of developing moderate‐severe disability (as assessed using Patient Determined Disease Steps (PDDS) (MS disease course not specified)). However, a cross‐sectional study in a similar UK cohort of SPMS of prematurely achieved vascular risk measured using Q‐RISK3 did not demonstrate any association with physical disability measures [29].

In this study, increased comorbidity burden also increased the risk of disability progression over 48 and 96 weeks where having 2 or more comorbidities was associated with an EDSS score that was 0.21 standard deviations worse over 48 and 96 weeks.

Our findings are in keeping with multiple studies that have investigated the risk of disability progression in those with an increasing number of comorbidities. When using prediction tools that combine vascular risk factors into a risk score, Moccia et al. (*n* = 251, median EDSS 3.5, MS subtype not specified) showed that a 1 percentage increase in Framingham risk score was associated with a 2.7 times increased EDSS score at follow up [31]. Whilst, Zhang et al. showed that each addition of a comorbidity increased mean EDSS by 0.18 in a longitudinal study of prospectively collected data in a predominantly relapse‐onset MS cohort (*n* = 3166, PPMS 8.6%) [26]. Our findings from a prospectively followed UK cohort from a randomised control trial build upon these findings, extending the concept that an increasing comorbidity burden is associated with physical disability progression even once in the secondary progressive phase of the disease.

T2 lesion volume was also strongly associated with increasing EDSS scores over 96 weeks, a known association linking white matter lesion load and disability in MS. However, T2LV in this cohort may also represent an excess burden of chronic small vessel disease which may also contribute to EDSS worsening in SPMS [32].

### 4.6 Strengths and Limitations

The strengths of our paper are the recruitment of a cohort with SPMS across many regions in the United Kingdom that included the systematic recording of VCM at baseline. Participants were assessed prospectively using blinded raters and standardised measures of physical and cognitive performance over a period of 48/96 weeks.

There are, however, several considerations. First, we relied on patient histories when recording comorbidities; however, we attempted to corroborate this with GP and hospital records where possible. As outlined in our previous paper in the journal, when examining hypertension, baseline blood pressure measurements were higher in those with hypertension (mean arterial pressure: 105 vs. 97, *p* < 0.001). Blood pressure was also higher across the study duration (mean MAP 102 vs. 95, *p* < 0.001, taken as an average across measurements from screening, 48‐ and 96 weeks). Second, we did not include the development of VCM during the trial period in this study, as we were more interested in the association between baseline VCM and longitudinal outcomes. Third, we recorded specific information on the two VCM listed, but it may have been of interest to include a wider range of VCM including diabetes mellitus, smoking history and previous vascular events. Fourth, we were unable to adjust for socio‐economic status as this data was not acquired as part of MS‐SMART [13, 14]. Fifth, amiloride has an anti‐hypertensive effect, and whilst treatment allocation was not significant in any of the 48/96‐week models, it may have reduced or affected our ability to detect an association between the presence of hypertension and disability progression over 48/96 weeks. Finally, the study period was limited to 2 years.

In summary, hypertension, hyperlipidaemia and comorbidity burden are associated with increased disability in SPMS, strengthening avenues for further intervention.

## Author Contributions

**Nevin A. John:** conceptualization, investigation, writing – original draft, methodology, validation, writing – review and editing, formal analysis, project administration, data curation, supervision. **Yingtong Li:** formal analysis; writing – writing results, sensitivity analysis; review and editing. **Floriana De Angelis:** data curation; formal analysis; resources; writing – review and editing; visualization. **Ferran Prados Carrasco:** methodology; software; data curation; investigation; formal analysis; project administration; writing – review and editing. **Jon Stutters:** software; data curation; project administration; resources; writing – review and editing. **Anisha Doshi:** Data curation; writing – review and editing. **Alberto Calvi:** data curation; writing – review and editing. **Domenico Plantone:** data curation; writing – review and editing. **Thomas Williams:** data curation; writing – review and editing. **Thanh Phan:** Formal analysis; writing – review and editing; supervision. **Jeremy Chataway:** conceptualization; methodology; funding acquisition; supervision; writing – review and editing.

## Funding

This work was supported by Efficacy and Mechanism Evaluation Programme.

## Conflicts of Interest

F.D.A., J.S., F.P.C., A.C., Y.L., T.W., D.P., A.D., and T.P. have no conflicts of interest to declare. N.A.J. is a local principal investigator on commercial MS studies funded by Novartis, Roche and Sanofi. He has received speakers' honoraria and consulting fees from Merck. He has received consulting fees, congress sponsorship covering registration and travel from Novartis. J.C. has received support from the Health Technology Assessment (HTA) Programme (National Institute for Health Research, NIHR), the UK MS Society, the US National MS Society and the Rosetrees Trust. He is supported in part by the National Institute for Health and Care Research, University College London Hospitals (UCLH), Biomedical Research Centre, London, UK. He has been a local principal investigator for a trial in MS funded by MS Canada. A local principal investigator for commercial trials funded by Ionis and Roche; and has taken part in advisory boards/consultancy for Biogen, Contineum Therapeutics, InnoCare, Lucid, Merck, NervGen, Novartis and Roche.

## Supporting information


**Data S1:** Supporting Information.

## Data Availability

The dataset from this study is held securely by University College London. Access may be granted via authorisation from the Executive Committee and may be facilitated by contacting NJ.
