# Supplementary material for: Vascular Comorbidities and an Increased Comorbidity Score Are Associated With Disability and Disability Progression in Secondary Progressive Multiple Sclerosis
Source: Eur J Neurol. 2026 Feb 9;33(2):e70517. doi: 10.1111/ene.70517 (PMC12886752; doi:10.1111/ene.70517)
Supplement: Supplementary file 1 — Data S1: Supporting Information. [file ENE-33-e70517-s001.docx]

**Supplementary Appendix**

**eTable 1. Association between hyperlipidaemia and EDSS change over 48/96-weeks, sensitivity analysis, with time interaction**

| Predictors | Beta | standardized 95% CI | p |
| --- | --- | --- | --- |
| Hyperlipidaemia | 0.18 | -0.03 – 0.39 | 0.088 |
| Hyperlipidaemia × Time | 0.08 | -0.21 – 0.37 | 0.597 |
| Other ethnicity | 0.45 | 0.02 – 0.87 | **0.039** |
| T2 lesion volume | 0.10 | 0.02 – 0.18 | **0.011** |
| Disease duration | -0.08 | -0.15 – -0.01 | **0.041** |

*n = 362. Other non-significant model covariates - age, sex, normalised brain volume, body mass index and treatment allocation are not shown

**eTable 2. Association between hyperlipidaemia and EDSS change over 48/96-weeks, sensitivity analysis, excluding amiloride group**

| **Predictors** | **Beta** | **Standardised 95% CI** | **p** |
| --- | --- | --- | --- |
| Hyperlipidaemia | 0.29 | 0.03 – 0.56 | **0.032** |
| Other ethnicity | 0.52 | 0.06 – 0.98 | **0.026** |
| T2 lesion volume | 0.13 | 0.03 – 0.23 | **0.010** |
| Disease duration | -0.07 | -0.15 – 0.01 | 0.101 |

*n = 268. Other non-significant model covariates – age, sex, normalised brain volume, disease duration, body mass index and treatment allocation are not shown

**eTable 3. Association between hyperlipidaemia and EDSS change over 48/96-weeks, sensitivity analysis, without T2 lesion volume**

| Predictors | Beta | standardized 95% CI | p |
| --- | --- | --- | --- |
| Hyperlipidaemia | 0.20 | -0.01 – 0.40 | 0.052 |
| Other ethnicity | 0.38 | -0.05 – 0.82 | 0.085 |
| Disease duration | -0.07 | -0.14 – 0.01 | 0.074 |

*n = 362. Other non-significant model covariates - age, sex, normalised brain volume, body mass index and treatment allocation are not shown

**eTable 4. Association between hyperlipidaemia and EDSS change over 48/96-weeks, sensitivity analysis, with study site**

| Predictors | Beta | standardized 95% CI | p |
| --- | --- | --- | --- |
| Hyperlipidaemia | 0.19 | -0.01 – 0.40 | 0.069 |
| Other ethnicity | 0.40 | -0.12 – 0.92 | 0.129 |
| White ethnicity | -0.45 | -0.85 – -0.05 | **0.028** |
| T2 lesion volume | 0.08 | 0.01 – 0.16 | **0.043** |
| Disease duration | -0.06 | -0.13 – 0.02 | 0.141 |

*n = 362. Other non-significant model covariates - age, sex, normalised brain volume, body mass index, treatment allocation and study site are not shown

**eTable 5. Association between comorbidity count and timed 25Ft walk at baseline**

| Predictors | Beta | Standardized 95% CI | p |
| --- | --- | --- | --- |
| One comorbidity | 0.07 | -0.14 – 0.29 | 0.515 |
| Two or more comorbidities | -0.40 | -0.70 – -0.10 | **0.009** |
| Sex | 0.29 | 0.08 – 0.49 | **0.006** |
| Black ethnicity | -0.14 | -0.24 – -0.04 | **0.007** |
| Other ethnicity | -0.88 | -1.50 – -0.26 | **0.006** |
| Normalised whole brain volume (mL) | -0.13 | -0.23 – -0.03 | **0.008** |

*n = 431, R^2^= 0.13. Non-significant model covariates – age, disease duration, T2 lesion volume, and body mass index are not shown

**eTable 6. Association between comorbidity count and EDSS change over 48/96-weeks, sensitivity analysis, with time interaction**

| Predictors | Beta | Standardized 95% CI | p |
| --- | --- | --- | --- |
| One comorbidity | 0.02 | -0.15 – 0.18 | 0.846 |
| Two or more comorbidities | 0.18 | -0.01 – 0.36 | 0.063 |
| One comorbidity × Time | 0.00 | -0.22 – 0.22 | 0.979 |
| > 2 comorbidities × Time | 0.07 | -0.13 – 0.27 | 0.475 |
| Other ethnicity | 0.45 | 0.03 – 0.87 | **0.036** |
| T2 lesion volume | 0.10 | 0.02 – 0.18 | **0.011** |
| Disease duration | -0.08 | -0.15 – -0.01 | **0.044** |

n = 362. Non-significant model covariates – age, sex, treatment allocation, normalised whole brain volume, and body mass index are not shown

**eTable 7. Association between comorbidity count and EDSS change over 48/96-weeks, sensitivity analysis, excluding amiloride group**

| **Predictors** | **Beta** | **Standardised 95% CI** | **p** |
| --- | --- | --- | --- |
| One comorbidity | 0.13 | -0.05 – 0.31 | 0.154 |
| Two or more comorbidities | 0.36 | 0.10 – 0.62 | **0.006** |
| Other ethnicity | 0.55 | 0.09 – 1.02 | **0.020** |
| T2 lesion volume | 0.13 | 0.03 – 0.23 | **0.012** |
| Disease duration | -0.07 | -0.16 – 0.01 | 0.079 |

*n = 268. Other non-significant model covariates – age, sex, normalised brain volume, disease duration, body mass index and treatment allocation are not shown

**eTable 8. Association between comorbidity count and EDSS change over 48/96-weeks, sensitivity analysis, without T2 lesion volume**

| Predictors | Beta | Standardized 95% CI | p |
| --- | --- | --- | --- |
| One comorbidity | 0.02 | -0.14 – 0.19 | 0.780 |
| Two or more comorbidities | 0.19 | -0.01 – 0.39 | 0.058 |
| Other ethnicity | 0.39 | -0.04 – 0.82 | 0.078 |
| Disease duration | -0.07 | -0.14 – 0.01 | 0.077 |

n = 362. Non-significant model covariates – age, sex, treatment allocation, normalised whole brain volume, and body mass index are not shown

**eTable 9. Association between comorbidity count and EDSS change over 48/96-weeks, sensitivity analysis, with study site**

| Predictors | Beta | Standardized 95% CI | p |
| --- | --- | --- | --- |
| One comorbidity | 0.02 | -0.14 – 0.19 | 0.776 |
| Two or more comorbidities | 0.20 | 0.01 – 0.40 | **0.040** |
| Other ethnicity | 0.41 | -0.11 – 0.93 | 0.126 |
| White ethnicity | -0.45 | -0.86 – -0.04 | **0.031** |
| T2 lesion volume | 0.08 | 0.01 – 0.16 | **0.043** |
| Disease duration | -0.06 | -0.13 – 0.02 | 0.144 |

n = 362. Non-significant model covariates – age, sex, treatment allocation, normalised whole brain volume, body mass index and study site are not shown


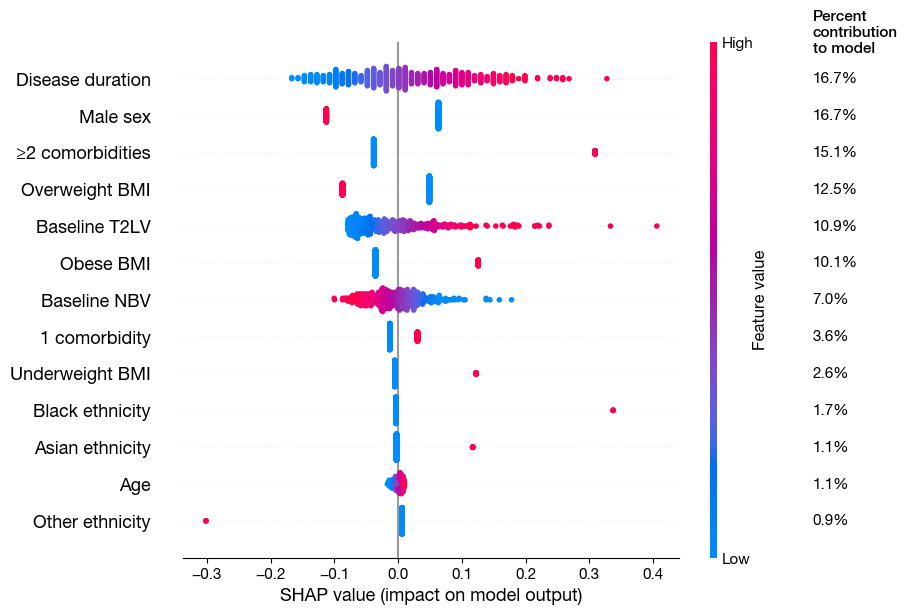


**eFigure 1. SHAP analysis of association between comorbidity count and baseline timed 25 foot walk**

SHAP values for parameters in the multiple linear regression evaluating the association between comorbidity count and baseline timed 25 foot walk. The column on the right shows the mean absolute SHAP value for each parameter, as a percentage of the total, representing the percent contribution of each parameter to the model output. The x-axis shows the SHAP values with each individual dot signifying SHAP value of a particular feature for a given data point.

BMI: Body mass index, NBV: normalized brain volume, SHAP: SHapley Additive exPlanations, T2LV: T2 lesion volume.
